# Gentamicin Augments the Quorum Quenching Potential of Cinnamaldehyde *In Vitro* and Protects *Caenorhabditis elegans* From *Pseudomonas aeruginosa* Infection

**DOI:** 10.3389/fcimb.2022.899566

**Published:** 2022-06-15

**Authors:** Jatin Chadha, Ravi, Jogender Singh, Sanjay Chhibber, Kusum Harjai

**Affiliations:** ^1^ Department of Microbiology, Panjab University, Chandigarh, India; ^2^ Department of Biological Sciences, Indian Institute of Science Education and Research (IISER), Mohali, India

**Keywords:** *Pseudomonas aeruginosa*, quorum sensing, virulence, biofilm formation, cinnamaldehyde, *Caenorhabditis elegans*, quorum quenching, anti-virulence therapy

## Abstract

The quorum sensing (QS) circuitry of *Pseudomonas aeruginosa* represents an attractive target to attenuate bacterial virulence and antibiotic resistance. In this context, phytochemicals harboring anti-virulent properties have emerged as an alternative medicine to combat pseudomonal infections. Hence, this study was undertaken to investigate the synergistic effects and quorum quenching (QQ) potential of cinnamaldehyde (CiNN) in combination with gentamicin (GeN) against *P. aeruginosa*. The QQ activity of this novel combination was evaluated using a QS reporter strain and synergism was studied using chequerboard assays. Further, the genotypic and phenotypic expression of pseudomonal virulence factors was examined alongside biofilm formation. The combination of CiNN and GeN exhibited synergy and promising anti-QS activity. This drug combination was shown to suppress AHL production and downregulate the expression of critical QS genes in *P. aeruginosa* PAO1. Molecular docking revealed strong interactions between the QS receptors and CiNN, asserting its QQ potential. Bacterial motility was compromised along with a significant reduction in pyocyanin (72.3%), alginate (58.7%), rhamnolipid (33.6%), hemolysin (82.6%), protease (70.9%), and elastase (63.9%) production. The drug combination successfully eradicated preformed biofilms and inhibited biofilm formation by abrogating EPS production. Our findings suggest that although GeN alone could not attenuate QS, but was able to augment the anti-QS potential of CiNN. To validate our results using an infection model, we quantified the survival rates of *Caenorhabditis elegans* following PAO1 challenge. The combination significantly rescued *C. elegans* from PAO1 infection and improved its survival rate by 54% at 96 h. In summary, this study is the first to elucidate the mechanism behind the QQ prospects of CiNN (augmented in presence of GeN) by abrogating AHL production and increasing the survival rate of *C. elegans*, thereby highlighting its anti-virulent properties.

## 1 Introduction

Indiscriminate use and unregulated consumption of antibiotics have paved the way for the emergence of multidrug-resistant (MDR) bacterial pathogens worldwide (Chadha and Khullar, 2021). Among the challenging ESKAPE pathogens, *Pseudomonas aeruginosa* has emerged as an extraordinarily versatile and notorious pathogen associated with a wide range of nosocomial infections in immunocompromised patients with relatively high mortality rates (Kang et al., 2003). *P. aeruginosa* is highly invasive, toxigenic, and fosters an arsenal of diverse virulence factors regulated through the intricate quorum sensing (QS) systems: Las, Rhl, and Pqs *via* QS signaling molecules called acyl-homoserine lactones (AHLs) (Chadha et al., 2021b). The LasR/LasI and RhlR/RhlI QS systems regulate the synthesis and signal transduction *via* N-3-oxo-dodecanoyl-L-homoserine lactone (3-oxo-C12-HSL) and N-butyryl-L-homoserine lactone (C4-HSL) autoinducers, respectively. In contrast, the Pqs system is chemically distinct from the AHL-mediated Las and Rhl systems and modulated through a non-AHL signaling molecule called 2-heptyl-3-hydroxy-4-quinolone (García-Reyes et al., 2020). These molecules harmonize bacterial responses at the community level and synchronize the expression of virulence genes associated with the production of toxins (exotoxin A), host-damaging enzymes (alkaline protease, LasA protease, LasB elastase, phospholipases), toxic secondary metabolites (hydrogen cyanide, rhamnolipids, hemolysins), and resilient biofilms (Chadha et al., 2021b). Since QS functions as an auxiliary factor that promotes pseudomonal virulence, its abrogation using QS inhibitors may possibly minimize the risk of exerting any selection pressure for the emergence of anthropogenic resistance, a phenomenon commonly witnessed with conventional antibiotic therapy (Rasmussen and Givskov, 2006). The fascinating property of a potent quorum quenching (QQ) agent is that it attenuates QS circuits of a pathogen at sub-inhibitory concentrations (sub-MICs) by either degrading the QS molecule or by abrogating its interaction with their cognate receptors without directly targeting the vital pathways required for bacterial survival (Kumar et al., 2021). Since this strategy does not impose any survival threat, the probability of developing resistance against the anti-QS drugs also remains low. Additionally, the spurring fidelity between the development of novel antibiotics, their safety approvals, and the evolution of drug resistance is inclined towards the latter end (Chadha, 2021). This has urged scientists and researchers to seek alternative therapy that attenuates pathogen virulence without hindering microbial growth. Hence, a “disarm-don’t kill” antivirulence approach can be revitalized to develop robust, effective, and targeted therapies against *P. aeruginosa*.

Although numerous investigations have identified novel QQ compounds, secondary plant metabolites and bioactive phytochemicals have recently gained attention from the scientific community owing to their anti-QS and anti-biofilm prospects (Chadha et al., 2021a). Their antimicrobial properties have been favored worldwide due to their natural existence, easy and economic availability, formidable pharmacological properties, and negligible cytotoxicity (Chadha et al., 2021c). Essential oils (EOs) contain a plethora of volatile secondary metabolites, including aldehydes, flavonoids, terpenoids, phenolics, polyacetylenes, and quinones that exhibit QQ at lower concentrations and antimicrobial properties at higher concentrations (Kumar et al., 2021). Cinnamon, derived from the inner bark of *Cinnamomum verum* has been long known as a traditional Indian spice and is used habitually in the Asian subcontinent. Cinnamaldehyde (CiNN), the principal bioactive and therapeutic phenylpropanoid present in cinnamon oil (EO), constitutes up to 70% (Mayaud et al., 2008), exhibiting remarkable antimicrobial, antitumor, anti-inflammatory, cardioprotective, and anti-QS properties (Doyle and Stephens, 2019). Reports documenting the role of CiNN in attenuating QS circuits and virulence factors in *P. aeruginosa* and *P. fluorescens* have surfaced recently (Li et al., 2018; Topa et al., 2020). Nevertheless, the anti-QS prospects of CiNN need further scrutiny to well elucidate its anti-virulent potential, mechanism of QS inhibition, and anti-fouling activity against *P. aeruginosa*. Therefore, the primary objective of this study was to examine the anti-virulent potential of CiNN in terms of phenotypic and genotypic expression of virulence factors and biofilm formation against *P. aeruginosa*, along with an antibiotic, gentamicin (GeN) *in vitro* and to further validate it using molecular docking and worm survival in a *C. elegans* infection model. Hence, we present data that demonstrates the QQ potential and anti-virulent properties of CiNN comprehensively using *in vitro*, *in silico*, and *in vivo* approaches.

## 2 Materials and Methods

### 2.1 Bacterial Strains, Growth Conditions, and Chemicals

The standard strain, *P. aeruginosa* PAO1 was obtained from Dr. Barbara H. Iglweski, Department of Microbiology and Immunology, University of Rochester (NY, USA). The AHL-deficient biosensor strain *Agrobacterium tumefaciens* NTL4 (pZLR4) was obtained from Dr. Jo Handelsman, Wisconsin University (WI, USA). *Escherichia coli* OP50 was obtained from the *Caenorhabditis* Genetics Centre (CGC), Minneapolis, USA. Unless otherwise indicated, all cultures were grown in Luria Bertani (LB) broth (HiMedia Labs, Mumbai, India) and stored in glycerol (50%) at -80°C. CiNN and GeN were purchased from Sigma-Aldrich (MO, USA) and HiMedia Labs (Mumbai, India), respectively. Working solutions of CiNN and GeN were prepared in 50% methanol (MeOH) and sterile distilled water, respectively.

### 2.2 Determination of Minimum Inhibitory Concentration (MIC)

MICs of CiNN and GeN were determined against *P. aeruginosa* PAO1 by microbroth dilution method using previously established protocols following the standard Clinical and Laboratory Standards Institute (CLSI) guidelines (Nascente et al., 2009). Briefly, two-fold serial dilutions of the test compounds were prepared in Muller Hinton (MH) broth in a 96 well microtiter plate. An inoculum containing 1× 10^8^ CFU/ml was added to every well, and the plate was incubated at 37°C for 24 h. Wells containing only media (LB) served as negative controls, while those lacking the test agents were employed as positive growth controls. Post incubation, 20 μl of 0.01% resazurin dye (Sigma-Aldrich, MO, USA) was added to every well and incubated at 37°C for 2 h. The lowest drug concentration that failed to produce a colour change, i.e., prevented resazurin reduction, was regarded as the MIC.

### 2.3 Analysis of Growth Curve

To select drug concentrations for subsequent investigations, *P. aeruginosa* PAO1 was grown in the presence of CiNN and GeN sub-MICs as described elsewhere (Bose et al., 2020). LB medium supplemented with CiNN and GeN at sub-MICs (1/2, 1/4, 1/8, and 1/16 MIC) was inoculated with overnight PAO1 culture at 1% and incubated at 37°C with shaking at 200 rpm. LB medium inoculated with PAO1 served as positive growth control, while LB supplemented with CiNN and GeN at MICs served as negative controls. Aliquots were drawn every 2 h from the growing cultures, and the growth profile was monitored by the recording changes in the A_600_ over a 24 h period. The drug concentration that did not retard or hinder bacterial growth was selected as the sub-MIC for further studies.

### 2.4 Testing the Drug Interactions *In Vitro*

#### 2.4.1 Chequerboard Dilution Assay

A standard chequerboard dilution assay was performed to determine the interaction between CiNN and GeN (Costa et al., 2019). Sterile double strength MH broth was dispensed to all the wells of a 96 well plate. Serial dilutions of CiNN and GeN were prepared in horizontal and vertical orientations of the microtiter plate starting from their 2X MICs. Early-log phase culture of *P. aeruginosa* PAO1 containing 1× 10^8^ CFU/ml was added to each well, and the plate was incubated at 37°C for 24 h. Wells containing only MH broth served as negative control, while those lacking the test agents were employed as positive growth control. Bacterial growth/inhibition was determined by adding resazurin dye as described previously. Fractional inhibitory concentration (FIC) index was calculated to examine the combinational effect of the drugs using the following formula: FIC of drug = MIC in combination/MIC alone, FIC index = FIC of CiNN + FIC of GeN. For a synergistic interaction, the FIC index was taken as ≤0.5. For additive, the FIC index was taken 0.5 ≤ FIC ≤ 1.0, 1.0 ≤ FIC ≤ 2.0 for indifferent, and for antagonism, the FIC index was more than 4.0.

#### 2.4.2 Time-Kill Assay

To confirm the synergistic interaction between CiNN and GeN, a time-kill assay was performed with *P. aeruginosa* PAO1 (Sopirala et al., 2010). Briefly, LB medium supplemented with CiNN (7.5μg/ml) and GeN (0.25μg/ml), alone and in combination, was inoculated using an overnight grown culture of PAO1 to achieve an initial cell density of 1× 10^6^ CFU/ml. LB medium lacking any drug(s) served as positive growth control. Cultures were allowed to grow at 37°C with shaking at 200 rpm. Aliquots were collected at different time intervals from the growing cultures. Their respective serial dilutions were spread-plated onto MacConkey agar and Cetrimide agar and incubated at 37°C for 24 h to selectively permit the growth of *P. aeruginosa*. Post incubation, the total bacterial count was calculated for every time interval in terms of log_10_ CFU/ml and plotted against the incubation time. The drugs were said to act in synergy if the combinational treatment reduced the bacterial count by a factor of 2 log_10_ or more after 24 h of incubation.

### 2.5 Extraction of AHLs and Bioassay for Anti-QS Activity

An overnight grown culture of *P. aeruginosa* PAO1 was used to isolate AHLs by solvent extraction method (Bose et al., 2020). Equal volumes of the cell-free supernatant (PAO1 culture) and acidified ethyl acetate were mixed, shaken vigorously, and incubated overnight at 4°C for the extraction of AHL molecules. Following incubation, AHLs were concentrated by evaporating the organic layer of ethyl acetate under reduced pressure using Rotavapor^®^ R-100 (Buchi, Flawil, Switzerland). The anti-QS activity of CiNN was then investigated by a qualitative assay employing the *A. tumefaciens* NTL4 biosensor strain with minor modifications (Bose et al., 2020). Briefly, 100 μl of X-gal (8 mg/ml in dimethylformamide), followed by 100 μl of extracted AHLs, were spread-plated onto an LB agar plate and allowed to dry. Then, 100 μl of *A. tumefaciens* NTL4 was evenly swabbed onto the agar surface. Wells were punched onto the plate using a sterile borer and 100 μl of the test agents, alone and in combination, were dispensed into the wells. The plate was incubated at 30°C for 24 h, and the zones of colourless halo around the wells were measured to assess the QQ activity. Well containing 50% MeOH served as a negative control.

### 2.6 Qualitative Assessment of AHL Production

The effect of sub-MICs of CiNN and GeN, alone and in combination on AHL production in PAO1 was examined by the thin-layer chromatography (TLC) overlay method with minor modifications (Gui et al., 2018). Briefly, AHLs were extracted and concentrated from cell-free supernatants of drug-treated and –untreated cultures. A reverse-phase TLC plate (TLC aluminium sheets 20 cm × 20 cm, RP-18 F254S, Merck, Darmstadt, Germany) of size 6 cm x 6 cm was cut to which synthetic AHL standards (2 μl each) and extracted AHLs (10 μl each) were applied and developed in 60:40 (v/v) of MeOH/water until the solvent front reached the top. The TLC plate was allowed to air dry for 30 min in a sterile container; meanwhile an agar cover layer of *A. tumefaciens* NTL4 was prepared. To 20 ml of molten soft agar (0.78% agar), 0.5 ml of X-gal (8 mg/ml), and 10 ml of overnight *A. tumefaciens* NTL4 culture were added, mixed well by shaking, and poured immediately onto the TLC plate. The overlaid TLC plate was incubated at 30°C for 48 h and the development of blue colouration was examined post-incubation. The AHL standards included: C4-HSL, C8-HSL, and 3-oxo-C12-HSL.

### 2.7 β-Galactosidase Assay for AHLs Quantification

AHL production in drug-treated and –untreated cell-free supernatants was determined using a modified β-galactosidase assay (Kumar et al., 2011). Briefly, 20 ml of *A. tumefaciens* NTL4 culture was centrifuged at 10,000 x *g* for 15 min and the cell pellet was washed twice with cold Z-buffer (0.06M Na_2_HPO_4_, 0.04M NaH_2_PO_4_, 0.01M KCl, 0.001M MgSO_4_; pH 7.0). The cell pellet was resuspended in sterile LB (20 ml) and 5 ml of extracted AHLs were added to this, followed by incubation at 30°C for 6 h with mild shaking. After incubation, the cells were centrifuged and the pellet was resuspended in 20 ml of cold Z-buffer. The absorbance value of cell suspension was recorded at 600 nm. Further, the cell suspension (1 ml) was lysed by adding 100 μl each of 0.1% SDS and chloroform, followed by vortexing for 1 min. To the cell lysate, 400 μl of o-nitrophenyl-D-galactopyranoside (ONPG; 4 mg/ml) was added and the reaction mixture was incubated at 30°C for 1 h. Post incubation, the enzymatic reaction was stopped by adding 1 ml of 1 M Na_2_CO_3_ and the absorbance value was measured at 420 nm. AHL production was determined in terms of β-galactosidase using the following formula:


$$\begin{array}{ll} \text{β}-\text{galactosidase}\;\text{activity}\;\left(\text{in Miller Units}\right)\;=\;{\text{A}}_{420\text{nm}}\times 1000/\;\text{Time}\;\left(\text{mins}\right)\times \text{Volume of culture}\;\left(\text{ml}\right) & \times A_{600\text{nm}}. \end{array}$$

### 2.8 RNA Isolation and Quantitative RT-PCR

Since CiNN showed promising anti-QS activity and subsequently inhibited the production of virulence factors in *P. aeruginosa* PAO1, qRT-PCR was employed to investigate the effect of CiNN on the transcript levels of different QS (*lasR*/*lasI* and *rhlR*/*rhlI*) and virulence genes (*aprA*, *plcH*, *rhlAB*, and *toxA*). *P. aeruginosa* PAO1 cultures were grown for 12 h in the presence and absence of CiNN and GeN sub-MICs, alone and in combination. Bacterial cells were harvested by centrifugation at 10,000 x *g* for 10 min at 4°C followed by washing with DEPC-treated water (0.1%). Total RNA was isolated from treated and untreated cells using Tri-Xtract™ reagent (G-Biosciences, MO, USA) according to the manufacturer’s instructions. The integrity of RNA was detected using a denaturing-agarose gel. First-strand cDNA was synthesized using iScript Reverse Transcription kit (Bio-Rad, USA) as per the manufacturer’s protocol. To quantify the expression of QS and virulence genes, qPCR was performed using iQ™ SYBR^®^ Green Supermix (Bio-Rad, CA, USA) with the primer pairs mentioned in **Table S1**. Glyceraldehyde-3-phosphate dehydrogenase (GAPDH) gene served as an internal control to normalize the expression levels of target genes. Reactions were performed in triplicates using 96-well PCR plates and 10 µl reaction volume. All reactions contained 1 µl of cDNA, 5 µl of SYBR Green Supermix, 0.5 µl of 10 µM of each primer and 3 µl of nuclease-free water. qRT-PCR amplifications were carried out using Thermocycler 8000 (Bio-Rad, USA). The relative gene expression was calculated using the 2^-∆∆Ct^ method and denoted in terms of fold change (Livak and Schmittgen, 2001).

### 2.9 Ligand Standardization and Molecular Docking Studies

The spatial coordinates of the QS proteins, LasR (PDB: 3IX3) and PqsR (PDB: 4JVC) were obtained from Protein Data Bank (PDB) (https://www.rcsb.org/) and the native ligand and water molecules were removed. The structure of RhlR was modeled since its experimental structure has not been elucidated yet. The protein sequence of RhlR (UniProtKB ID: P54292) was obtained from the UniProtKB database (http://www.uniprot.org/) and homology modeling was performed using Rosetta Comparative Modelling (Robetta Server). The model was validated using MolProbablity, Verify3D, and ERRAT servers based on stereochemical and geometric parameters analysis. The structures were subjected to energy minimization using GROMACS software suite (v2020.6) employing CharMM36 force field followed by the addition of Kollman charges and polar hydrogens using Autodock Tools. The docking grid was generated based on the active site defined by the residues listed in **Table S2**. The structures of ligands, CiNN (PubChem CID: 637511), furanone C-30 (PubChem CID: 10131246), 3-oxo-C12-HSL (PubChem CID: 3246941), and C4-HSL (PubChem CID: 443433) were retrieved from PubChem in sdf format and converted to 3D structures with polar hydrogens using Open Babel. Molecular docking was then performed using AutoDock Vina (Version 1.2.0) with exhaustiveness of ‘24’ and the best binding pose was selected based on predicted binding energy and molecular interactions. Docking conformations of each ligand were analyzed by examining their binding energy score (kcal/mol). The docking score of CiNN was compared with the natural ligand of each receptor.

### 2.10 Effect on Motility Phenotypes of *P. aeruginosa*

Sub-MIC treated and untreated cultures of PAO1 were used for investigating the effect of CiNN and GeN, alone and in combination, on the motility phenotypes of *P. aeruginosa* (Bose et al., 2020).

#### 2.10.1 Swimming Motility

*P. aeruginosa* PAO1 cultures were point inoculated onto media containing 1.0% tryptone, 0.5% NaCl, and 0.3% agar (supplemented with sub-MICs of CiNN and GeN) and incubated at 30°C for 24 h. Post incubation, the media plates were checked for swimming motility by measuring the radius of circular expansion around the point of bacterial inoculation.

#### 2.10.2 Swarming Motility

2.10.2 Nutrient agar plates containing 0.5% each of agar, D-glucose, NaCl, and supplemented with CiNN and GeN at sub-MICs were point inoculated with overnight grown cultures of *P. aeruginosa* PAO1. Swarming motility was determined by measuring turbid circular zones after incubation for 24 h at 37°C.

#### 2.10.3 Twitching Motility

LB agar plates (1% agar) containing CiNN and GeN at sub-MIC levels were stabbed with cultures of *P. aeruginosa* PAO1 using sterile toothpicks followed by incubation at 37°C for 24 h. The diameter of bacterial growth (interstitial zone) was then measured to assess the impact of drugs on the twitching motility of PAO1.

### 2.11 Effect on QS-Regulated Virulence Factors of *P. aeruginosa*

The QS-regulated virulence factors of *P. aeruginosa* PAO1 were assessed following treatment with sub-MICs of CiNN and GeN, alone and in combination, in the cell-free supernatant.

#### 2.11.1 Alginate Production

The alginate concentration was assessed in untreated and untreated culture supernatants with slight modifications (Mathee et al., 1999). Alginate in the supernatant was precipitated on an ice bath by adding an equal volume of 2% (w/v) cetylpyridium chloride followed by centrifugation at 10,000 x *g* for 10 min at 4°C. The alginate pellet thus obtained was suspended in 5 ml of ice-cold 1 M NaCl, then precipitated again with 5 ml of ice-cold isopropanol and centrifuged at 10,000 x *g* for 10 min at 4°C. The pellet obtained was resuspended in 1 ml of ice-cold 0.85% NaCl. Carbazole assay was used to quantify alginate by mixing 0.7 ml of the dissolved pellet with 1.3 ml of sulfuric acid/borate solution followed by vortexing and heating at 55°C for 20 min. The tubes were re-equilibrated in an ice bath for 10 min, and 0.2 ml of 0.1% carbazole solution (in EtOH) was added. The tubes were vortexed and incubated at room temperature (RT) for 1 h. The absorbance values were recorded at 530 nm and the alginate concentration (in mg/ml) was determined by extrapolating values from a standard curve of sodium alginate.

#### 2.11.2 Rhamnolipid Production

*P. aeruginosa* PAO1 was cultured in the presence and absence of CiNN and GeN sub-MICs, alone and in combination, in a chemically defined media as described previously (Bose et al., 2017). Cell-free supernatants were used for quantifying rhamnolipid production by a colorimetry-based orcinol-sulfuric acid assay (Cheng et al., 2017). Briefly, 1 ml of culture supernatant was mixed with 9 ml of 0.19% orcinol prepared in 53% sulfuric acid and incubated at 80°C for 30 min. The solutions were cooled at RT and the absorbance values were recorded at 420 nm. Rhamnolipid concentration (in μg/ml) was determined by normalizing the data to a standard curve of L-rhamnose. For the conversion of rhamnose to rhamnolipid, a factor of 2.5 was used based on the assumption that 1 μg of rhamnose corresponds to 2.5 μg of the rhamnolipid mixture (Bose et al., 2017).

#### 2.11.3 Hemolysin Production

The hemolytic potential of *P. aeruginosa* was assessed in cell-free supernatants as reported earlier (Rossignol et al., 2008). Sheep erythrocytes were washed thrice with cold saline (sterile) and resuspended in saline to a final concentration of 2%. For hemolysis assays, 0.8 ml of culture supernatant was mixed with 0.8 ml of the 2% erythrocyte suspension and incubated at 37°C for 2 h. Post incubation, the suspension was centrifuged at 10,000 x *g* for 10 min at 4°C, and hemoglobin release was assessed by measuring the absorbance values at 545 nm. The concentration of hemoglobin released (in mg/ml) was determined by normalizing the data to a standard curve of hemoglobin.

#### 2.11.4 Pyocyanin Production

The amount of pyocyanin produced in the culture supernatant was quantified by a chemical assay (Essar et al., 1990). Pyocyanin was extracted by adding 3 ml of chloroform to 5 ml of culture supernatant, followed by vigorous mixing and incubation at RT for 30 min. The chloroform layer was separated, mixed with 1 ml of 0.2 M HCl, and centrifuged at 5000 x *g* for 5 min. The upper red layer of HCl was separated and the absorbance was measured at 520 nm. The amount of pyocyanin produced (in μg/ml) was calculated by multiplying the A_520_ values by a factor of 17.072, as reported previously (Essar et al., 1990).

#### 2.11.5 Protease Production

Proteolytic activity in the culture supernatants was measured by a modified azocasein assay (Nicodeme et al., 2005). For the protease assay, 1 ml of culture supernatant was mixed with 1 ml of 10 mM Tris-Cl buffer (pH 7.5) containing azocasein (3 mg/ml) and 0.5 mM CaCl_2_. The reaction mixture was incubated at 37°C for 1 h with mild shaking. The reaction was stopped by adding 0.4 ml of 10% trichloroacetic acid, followed by centrifugation at 10,000 x *g* for 10 min and measuring the absorbance of the supernatant at 420nm. Specific Proteolytic activity Unit (U) was calculated by dividing the A_420_ values by the A_600_ (cell density) of the respective bacterial cultures.

#### 2.11.6 Elastase Production

The elastolytic activity was measured using a modified protocol employing elastin-congo red as the substrate (El-Mowafy et al., 2014). Briefly, 1 ml of culture supernatant was mixed with the 1 ml of elastin-congo red (20 mg/ml) in 10 mM Tris-Cl buffer (pH 7.5) supplemented with 0.5 mM CaCl_2_ and incubated at 37°C for 2 h with mild shaking. To this, 1 ml of 0.7 M sodium phosphate buffer (pH 6.0) was added to stop the reaction and A_495_ values were recorded following centrifugation at 10,000 x *g* for 10 min. Enzyme activity was expressed in elastolytic units (U) using the following formula: Elastolytic units (U) = Amount of congo red released (in μg)/Mol. Wt._Congo Red_ x Incubation time (in min).

### 2.12 Examining the Anti-Fouling Effect of CiNN and GeN

#### 2.12.1 Qualitative Assessment of Biofilm Formation

Preliminary experiments were undertaken to investigate the biofilm-forming capacity of *P. aeruginosa* PAO1 in the presence of CiNN and GeN sub-MICs, alone and in combination (Bose et al., 2020). Sterile polypropylene microcentrifuge tubes (Tarsons, Kolkata, India) containing 1.5 ml LB medium supplemented with drugs at sub-MICs were inoculated with 20 μl of an overnight grown culture of PAO1 and incubated at 37°C for 24 h under static conditions. Tube without drugs served as positive growth control. Post incubation, media containing loose planktonic cells was aspirated and tubes were washed with phosphate-buffered saline (PBS, pH 7.2) to remove any loosely bound cells. The adherent biofilms were then stained with 0.1% crystal violet (CV). After 15 min, tubes were washed thrice with PBS to remove excess dye, and the adherence scores (+: weak, ++: moderate, ++++: strong biofilm) were assigned based on the color intensity of adherent CV to assess biofilm formation qualitatively.

#### 2.12.2 Quantitative Biofilm Inhibition Assay

Biofilm inhibition assay was performed in the presence of CiNN and GeN sub-MICs, alone and in combination, in 96 well flat-bottom microtiter plates (Tarsons, Kolkata, India). Wells containing 5 ml LB medium supplemented with sub-MICs of the drug(s) were inoculated with a diluted culture of *P. aeruginosa* (1×10^6^ CFU/ml). The plate was incubated at 37°C under static conditions. Sterile LB was used as a negative control, while PAO1 culture without any drug treatment served as a positive control. The media containing planktonic cells was discarded; wells were washed thrice with PBS (pH 7.2), and the adherent biofilms were stained with 0.1% CV. After staining, the dye was removed, and wells were washed thrice with PBS (pH 7.2) to remove excess CV. The plate was air-dried at 37°C, followed by adding 33% glacial acetic acid to each well to elute the bound dye. Absorbance values were measured using an ELISA plate reader (Bio-Rad, Hercules, CA) at 595 nm. All sets were processed similarly for up to 7 days.

#### 2.12.3 Preformed Biofilm Eradication Assay

Overnight culture of PAO1 was diluted using sterile LB to attain 1×10^6^ CFU/ml and 200 μl of the adjusted culture was dispensed into a 96 well flat-bottom microtiter plate to allow biofilm formation. After every 24 h, the spent culture medium was discarded, and fresh LB was replenished in the wells. Cultures were allowed to grow at 37°C under static conditions for 1 day (young biofilm), 4 days (peak biofilm), and 7 days (mature biofilm). On completion of the designated time, preformed biofilms were challenged with sub-MICs of CiNN and GeN, alone and in combination, for 24 h at 37°C. Wells lacking the drugs served as positive control, while sterile LB was used as negative control. The contents were aspirated to remove any planktonic cells from the treated and untreated wells, followed by washing thrice with PBS. After washing, adherent biofilms were stained with 0.1% CV, followed by washing with PBS. The bound CV was then eluted from adherent biofilms using 33% acetic acid and the absorbance values were recorded at 595 nm.

#### 2.12.4 Microscopic Examination of *P. aeruginosa* Biofilms

Scanning electron microscopy (SEM) was employed to examine biofilm formation in PAO1 following drug treatment visually (Bose et al., 2020). PAO1 biofilms were allowed to form over sterile coverslips placed in 5 ml of LB medium supplemented with sub-MICs of CiNN and GeN, alone and in combination, in a six-well culture dish at 37°C for 4 days. PAO1 culture without any drug treatment served as a positive control. After every 24 h, the culture medium was discarded, and fresh LB containing the drug(s) was added to the wells. Post incubation, the spent media was aspirated and coverslips were washed with PBS (pH 7.2) to remove any unbound cells. Peak day biofilms were fixed with 2% glutaraldehyde for 2 h at room temperature, followed by three rounds of washing with PBS. Biofilms were then sequentially dehydrated using different concentrations of EtOH, including 30, 50, 70, 90, and 100%. Coverslips were air-dried, gold-coated, and viewed under the field emission scanning electron microscope (FESEM; SU8010, Hitachi, Tokyo, Japan) to analyze PAO1 biofilms.

### 2.13 *C. elegans* Strains and Slow Killing Assays With *P. aeruginosa* PAO1

Slow-killing (SK) assays were used to examine the protective (anti-virulence) effects of the drug combination as described elsewhere (Singh and Aballay, 2019). *C. elegans* wild-type Bristol N2 hermaphrodites were maintained on a lawn of *E. coli* OP50 at 20°C unless otherwise indicated. Gravid adults were transferred to *E. coli* OP50 lawns and allowed to lay eggs for 2 h. The gravid adults were removed, and the eggs were allowed to develop at 20°C to late L4s for subsequent assays. SK plates (modified NGM agar plate) supplemented with GeN and CiNN (alone and in combination) were used for the assay. PAO1 cultures were raised by inoculating individual bacterial colonies into 2 ml of LB broth and incubating them at 37°C with shaking for 10–12 h. To obtain a uniform bacterial lawn, 30 μl of PAO1 culture (A_600_ ~ 0.3) was spread-plated onto the different sets of SK plates: control (untreated PAO1), GeN alone, CiNN alone, and CiNN-GeN in combination. The plates were incubated at 37°C for 12 h and then cooled to room temperature for at least 30 min before seeding with thirty synchronized late L4 stage worms. The killing assays were performed at 25°C, and live animals were transferred daily to fresh plates. Animals were scored at times indicated and were considered dead when they failed to respond to touch.

### 2.14 Statistical Analysis

All experiments were performed in triplicates (biological), and the mean values ± standard deviations were calculated. Experiments were conducted in three independent events and a representative set of data has been reported. Significance of the data was evaluated with one-way analysis of variance (ANOVA) test using GraphPad Prism ver. 8.0. *p*-values of < 0.05 were considered to be statistically significant (p values: * ≤ 0.05, ** ≤ 0.01, *** ≤ 0.001, **** ≤ 0.0001).

## 3 Results

### 3.1 Effect of CiNN and GeN on the Growth of *P. aeruginosa*

**Figure 1:**
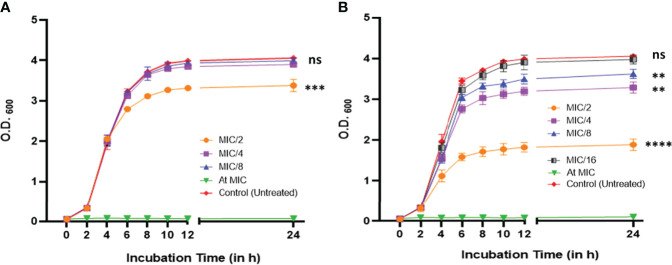
Growth profile of *P. aeruginosa* PAO1 at 600 nm in the absence and presence of different sub-minimum inhibitory concentrations of **(A)** CiNN and **(B)** GeN. (ns, not significant, **p ≤ 0.01, ***p ≤ 0.001, ****p ≤ 0.0001).

The MICs against PAO1 were found to be 30.0 μg/ml and 4.0 μg/ml for CiNN and GeN, respectively (**Supplementary Figure 1**). Subsequently, the growth curves of PAO1 at sub-MICs of CiNN and GeN demonstrated typical sigmoidal growth kinetics with distinguishable lag, log, and stationary phases of bacterial growth at 37°C. At 1/2 MIC, CiNN hindered bacterial growth with early saturation at 24 h, whereas other sub-MICs (1/4 and 1/8 MIC) exhibited growth profiles similar to that of untreated control (**Figure 1A**). In comparison, GeN showed a dose-dependent effect on PAO1 growth. Lower sub-MIC of GeN (1/16 MIC) did not significantly affect bacterial growth, but higher sub-MICs (1/2, 1/4, and 1/8 MIC) reduced bacterial growth rate with saturation at early stages (**Figure 1B**). Hence, further experiments were conducted at sub-MICs of both the drugs.

### 3.2 CiNN and GeN Exhibit Synergy Against *P. aeruginosa*

**Figure 2:**
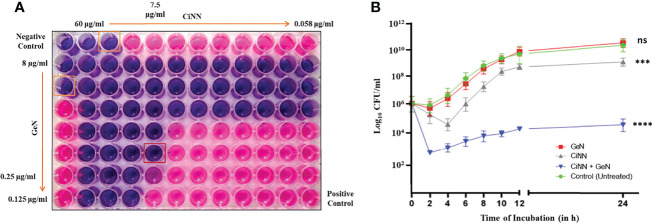
Evaluating the synergism between CiNN and GeN against *P. aeruginosa* PAO1 using **(A)** the standard chequerboard assay and **(B)** time-kill kinetics assay. (ns, not significant, ***p ≤ 0.001, ****p ≤ 0.0001).

A standard chequerboard dilution assay was employed to determine the interaction between CiNN and GeN against *P. aeruginosa* PAO1 *in vitro*. When tested in combination, the MICs of CiNN and GeN showed a four- and eightfold reduction, respectively (**Figure 2A**). The FIC index for the phytochemical-antibiotic combination was found to be 0.375 (**Table 1**), indicative of a strong synergistic interaction between CiNN and GeN against *P. aeruginosa* PAO1. These findings were further extended and confirmed using a time-kill kinetics assay. Growth of PAO1 in the presence of CiNN and GeN alone showed 1.53- and 0.94-log_10_ CFU/ml reduction, respectively, with reference to the untreated control. In contrast, combinational treatment with CiNN and GeN resulted in a 7.67 log_10_ CFU/ml reduction (**Figure 2B**), hence confirming synergistic interaction between the two drugs. Therefore, CiNN (7.5 μg/ml) and GeN (0.25 μg/ml) were used for subsequent investigations.

**Table 1:** Fractional inhibitory concentration (FIC) and FIC index for testing synergy between CiNN and GeN against *P. aeruginosa* PAO1.

Organism	Agent	MIC alone	MIC in Combination	FIC	FICI	Nature of Interaction
*P. aeruginosa* PAO1	Cinnamaldehyde	30.0 μg/ml	7.5 μg/ml	0.25	0.375	Synergy
	Gentamicin	4.0 μg/ml	0.25 μg/ml	0.125		

### 3.3 GeN Augments the Anti-QS Activity of CiNN and Reduces Production of AHL in *P. aeruginosa*

**Figure 3:**
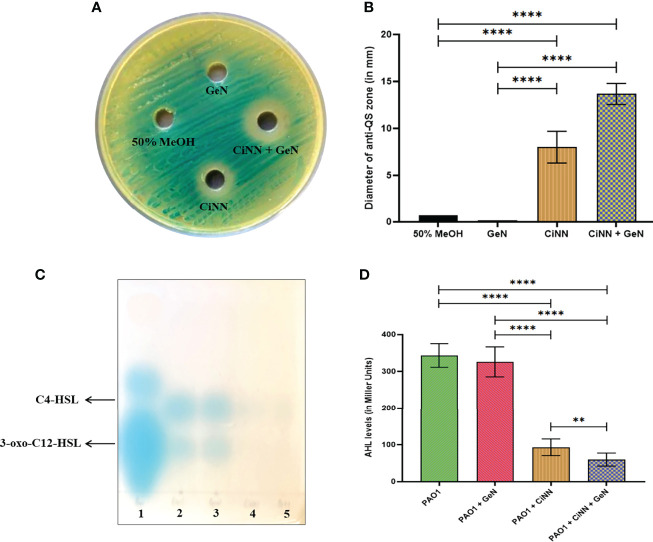
Analyzing the anti-QS activity of CiNN and GeN, alone and in combination. **(A)** Qualitative test showing the colorless zones surrounding the wells indicating the quorum quenching potential of CiNN employing *Agrobacterium tumefaciens* NTL4 biosensor strain. **(B)** Graph showing the diameter of anti-QS zones obtained with the test agents. **(C)** TLC agar overlay technique with AHLs. Lane 1: AHL standard containing C4-HSL, C8-HSL, and 3-oxo-C12-HSL, Lane 2: AHLs extracted from untreated PAO1 (Control), Lane 3: AHLs extracted from GeN-treated culture, Lane 4: AHLs extracted from CiNN-treated culture, Lane 5: AHLs extracted from CiNN-GeN-treated culture. **(D)** β-galactosidase assay for the quantification of AHL production in treated and untreated PAO1 cultures. (**p ≤ 0.01, ****p ≤ 0.0001).

The anti-QS activity of the drugs was assessed using the *A. tumefaciens* NTL4 biosensor strain capable of detecting long-chain AHLs. CiNN containing wells exhibited a colorless halo indicative of its anti-QS potential (**Figure 3A**), while wells supplemented with GeN did not show any such effects. Interestingly, wells loaded with CiNN and GeN in combination showed a notable rise in the QQ activity with a significant increase in the diameter of the colorless halo (**Figure 3B**). This led to speculation that the CiNN-GeN combination may be responsible for lowering the production of AHL molecules, thereby demonstrating increased anti-QS activity. Therefore, the agar overlay technique on TLC plates was used to examine AHL production in PAO1 cultures treated with CiNN and GeN, alone and in combination. As indicated from the colored spots on the chromatogram, AHL production was significantly reduced in the presence of CiNN and further lowered in PAO1 cultures grown in the presence of sub-MICs of CiNN and GeN in combination (**Figure 3C**). This substantiated our previous observations and we extended our findings by quantifying the production of AHL molecules in drug-treated and untreated cultures. AHL production in *P. aeruginosa* cultures was determined as a function of β-galactosidase activity (in Miller Units) resulting from AHL-mediated induction of *lacZ* on pZLR4. Our results indicated that treatment with CiNN significantly lowered the β-galactosidase activity by ~ 3.69 folds (93.1 MU), while the latter remained unaffected following exposure to GeN (326 MU) in comparison with that of the untreated control (343 MU) (**Figure 3D**). Moreover, combinational treatment with CiNN and GeN incurred a sharp decline in the enzyme activity by 5.73 folds (59.9 MU). This strongly points towards the role of GeN in enhancing the anti-QS prospects of CiNN by affecting AHL production in *P. aeruginosa*. Hence, this combination harbors profuse QQ activity that may prove to be effective in downregulating the QS-regulated virulence and biofilm formation in *P. aeruginosa*.

### 3.4 CiNN Downregulates the Expression of QS-Associated Genes in *P. aeruginosa*

**Figure 4:**
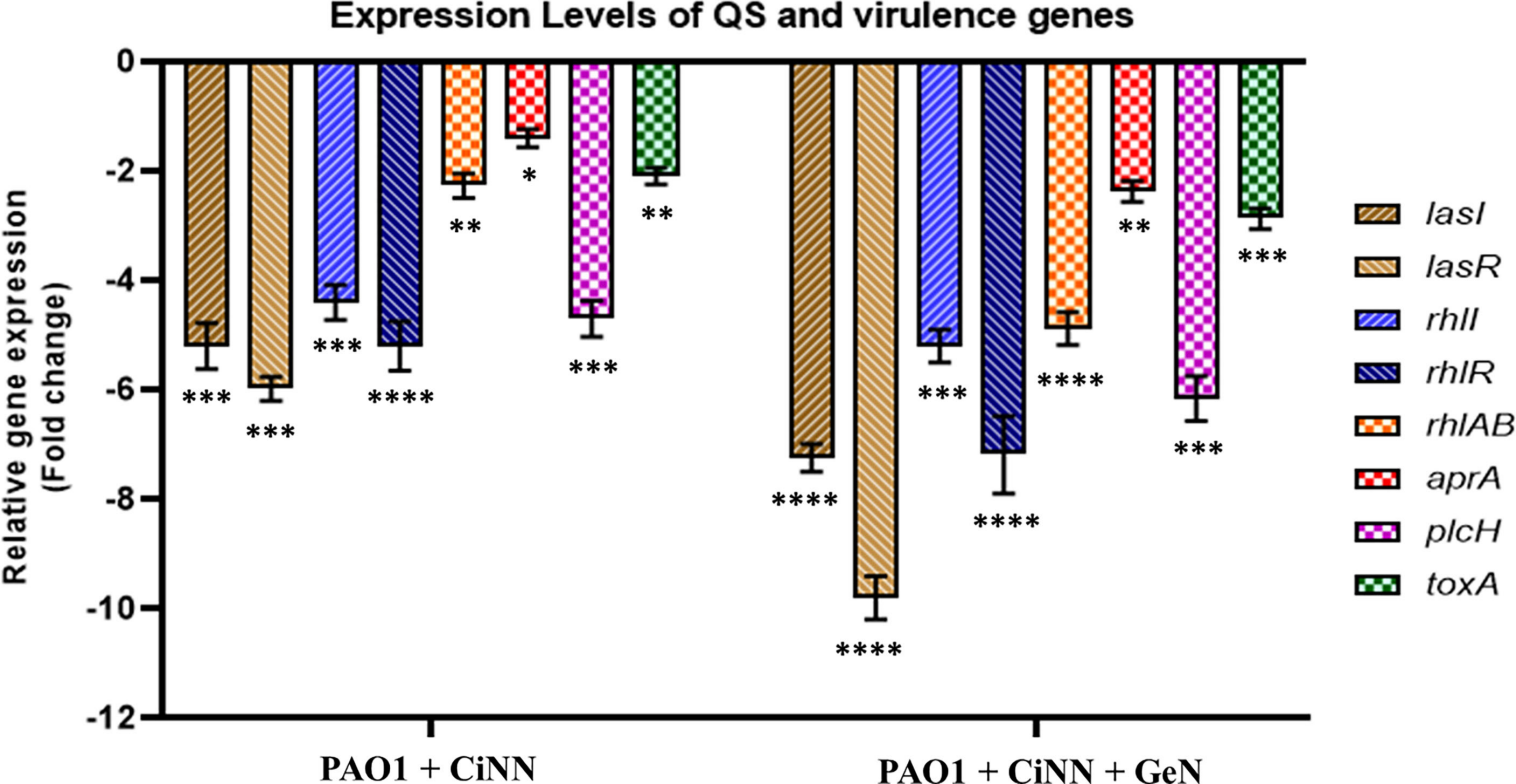
Relative expression of key QS-related genes (*lasI*, *lasR*, *rhlI*, and *rhlR*) and virulence genes (*rhlAB*, *aprA*, *lasB*, toxA, and *plcH*) associated with the production of virulence factors in *P. aeruginosa*, in the presence of CiNN alone and CiNN-GeN combination. Gene expression has been normalized with GAPDH and depicted relative to the expression of genes in the untreated control. (*p ≤ 0.1, **p ≤ 0.01, ***p ≤ 0.001, ****p ≤ 0.0001).

qRT-PCR was employed to determine the expression levels of various QS genes (*lasI*/*lasR*, *rhlI*/*rhlR*) and virulence-associated genes of PAO1 encoding rhamnolipids (*rhlAB*), exotoxin A (*toxA*), alkaline protease (*aprA*), and phospholipase C (*plcH*) following drug treatment, alone and in combination. CiNN alone significantly downregulated the expression of *lasI*, *lasR*, *rhlI*, and *rhlR* by 3.4-, 3.9-, 4.4-, and 5.2-folds, while combinational treatment with GeN exerted further repression over all the QS genes by 3.8-, 4.2-, 5.2-, and 7.2-folds, respectively (**Figure 4**). The QS-regulated virulence genes of *P. aeruginosa*, *aprA*, *plcH*, *rhlAB*, and *toxA* also showed notable downregulation upon CiNN exposure by 1.4-, 4.7-, 2.3-, and 2.1-folds, respectively. This repression was further augmented in the presence of GeN by 2.4-, 6.2-, 4.9-, and 3.7-folds, respectively, for the virulence genes (**Figure 4**). Taken together, our results strongly indicate that CiNN overrides the QS machinery by downregulating the expression of QS genes and production of AHL molecules, thereby silencing the virulence circuits of *P. aeruginosa*. In addition, GeN elevates the QQ activity of CiNN, enhancing the attenuation of QS circuitry and QS-regulated virulence in PAO1.

### 3.5 *In Silico* Analysis Reveals Strong Interaction of CiNN With QS Receptors of *P. aeruginosa*

To validate the anti-QS activity of CiNN virtually, we docked it with LasR, RhlR, and PqsR QS receptors of *P. aeruginosa*. We compared its binding energies with that of natural ligands (3-oxo-C12-HSL, C4-HSL, and PQS), respectively, and a known QS inhibitor, furanone C-30. The structure of RhlR was modeled and was found to be in agreement with the previously reported ones (Kumar et al., 2015; Bose et al., 2020). Molecular docking revealed the putative binding pose of CiNN with LasR, PqsR, and RhlR. CiNN displayed strongest interaction with the LasR receptor (-12.0 kcal/mol), followed by PqsR (-8.2 kcal/mol), and RhlR (-7.6 kcal/mol) (**Table S2**). All the QS receptors showed highly effective binding to their respective natural ligands (controls) and furanone C-30. The three-dimensional representations of docking interactions of CiNN and furanone C-30 have been depicted in **Supplementary Figures 2** and **3**, respectively. The docking results suggest that CiNN interacts with the QS receptors of *P. aeruginosa* majorly through hydrophobic interactions, as expected from its low TPSA value (17.07 Å²). In complex with LasR, CiNN formed van der Waals (VdW) interactions with eleven residues, a hydrogen bond (H-bond) with Ser129, two carbon-hydrogen (C–H) bonds with Tyr56 and Tyr64 residues, and one alkyl bond with Ala105 residue (**Supplementary Figure 2**). With the PqsR receptor, CiNN was predicted to form VdW interactions with twelve amino acid residues, a single H-bond with Thr166, three C-H bonds with Thr166, Ile236, and Ile263, and two alkyl bonds with Leu208 and Ile236 residues. Similar associations were observed with RhlR as it formed VdW interactions with thirteen residues, one H-bond with Ser135, and two alkyl bonds with Ala83 and Ala111 residues of RhlR ligand-binding site. CiNN was also speculated to form a Pi-sigma bond with the Trp96 residue (**Figure S2**). Additionally, CiNN displayed overlapping interactions with the natural ligands of the QS receptors and furanone C-30 (**Table S2**). Overall, these findings collectively establish the role of CiNN in attenuating QS circuits by associating with the QS receptors of *P. aeruginosa*.

### 3.6 CiNN in Combination With GeN Inhibits the Motility Phenotypes of *P. aeruginosa*

**Figure 5:**
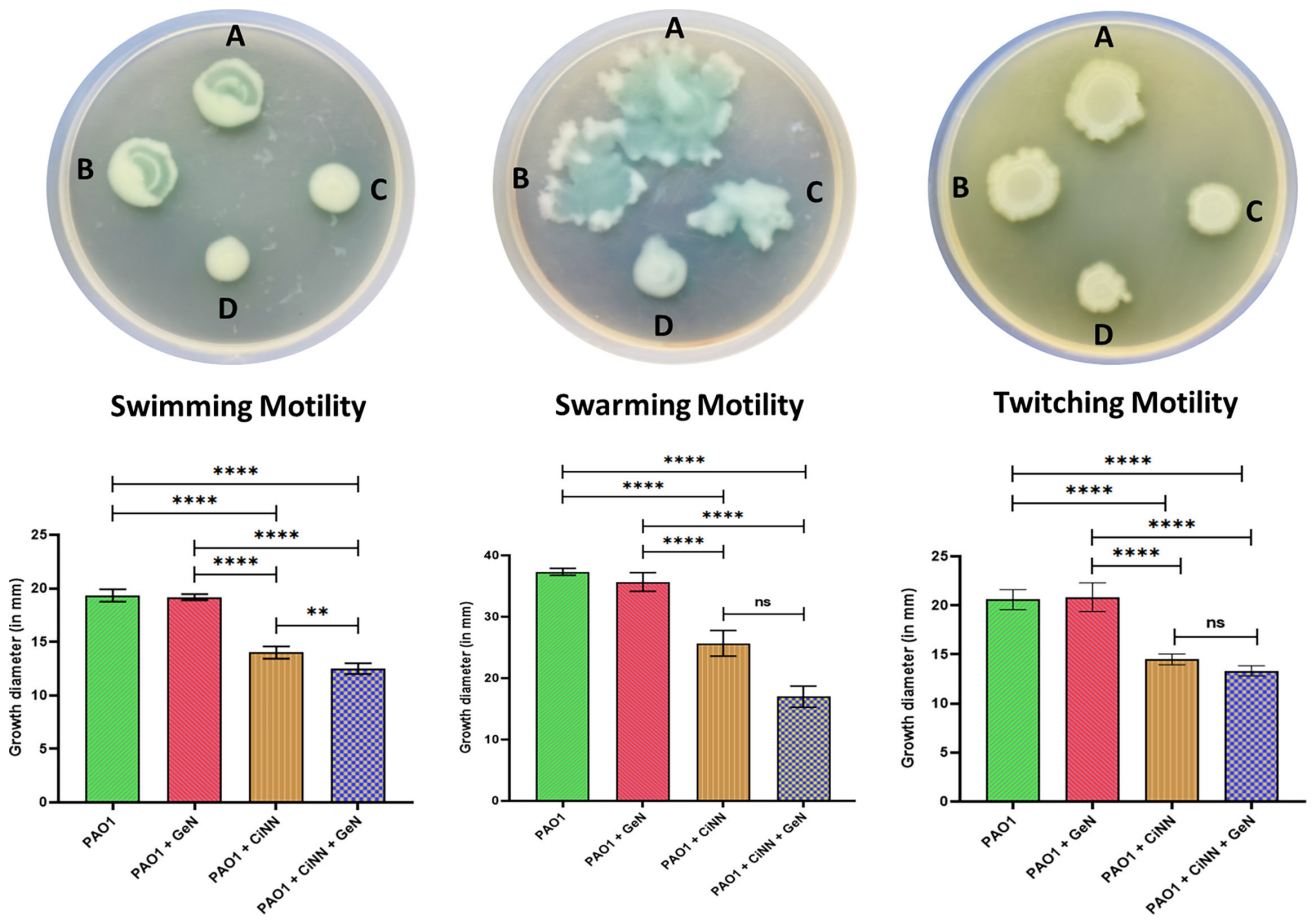
Anti-motility effect of the test drugs on *P. aeruginosa* PAO1. Upper panel: Bacterial growth on different motility media. **(A)** Untreated control, **(B)** GeN-treated culture, **(C)** CiNN-treated culture, and **(D)** CiNN-GeN-treated culture. Lower panel: Graphs depicting the diameters of bacterial growth corresponding to each motility media (swimming, swarming, and twitching motility). (ns, not significant, **p ≤ 0.01, ****p ≤ 0.0001).

Since the drug combination showed enhanced anti-QS activity, we speculated that it might hinder the motility phenotypes of *P. aeruginosa*. Hence, we examined these effects on swimming, swarming, and twitching motilities of *P. aeruginosa* PAO1 by point inoculating the drug-treated and –untreated cultures onto different motility media and recording the diameters of bacterial growth (**Figure 5**, upper panel). CiNN alone was shown to significantly inhibit the swimming, swarming, and twitching motilities of *P. aeruginosa* by 27.6%, 31.2%, and 29.5%, respectively, as compared to the untreated controls (19.34 mm, 37.34 mm, and 20.58 mm) (**Figure 5**, lower panel). Interestingly, combinational treatment with CiNN and GeN retarded all forms of pseudomonal motilities to a greater extent than CiNN alone (35.4%, 54.5%, and 35.2%, respectively). On the contrary, GeN failed to exert any inhibitory effects on the swimming, swarming, and twitching motility of PAO1 (19.17 mm, 35.67 mm, and 20.83 mm, respectively). Hence, the drug combination caused suppression of all forms of motility in *P. aeruginosa* PAO1.

### 3.7 CiNN and GeN Attenuate Pseudomonal Virulence by Impeding the Production of QS-Regulated Virulence Factors

**Figure 6:**
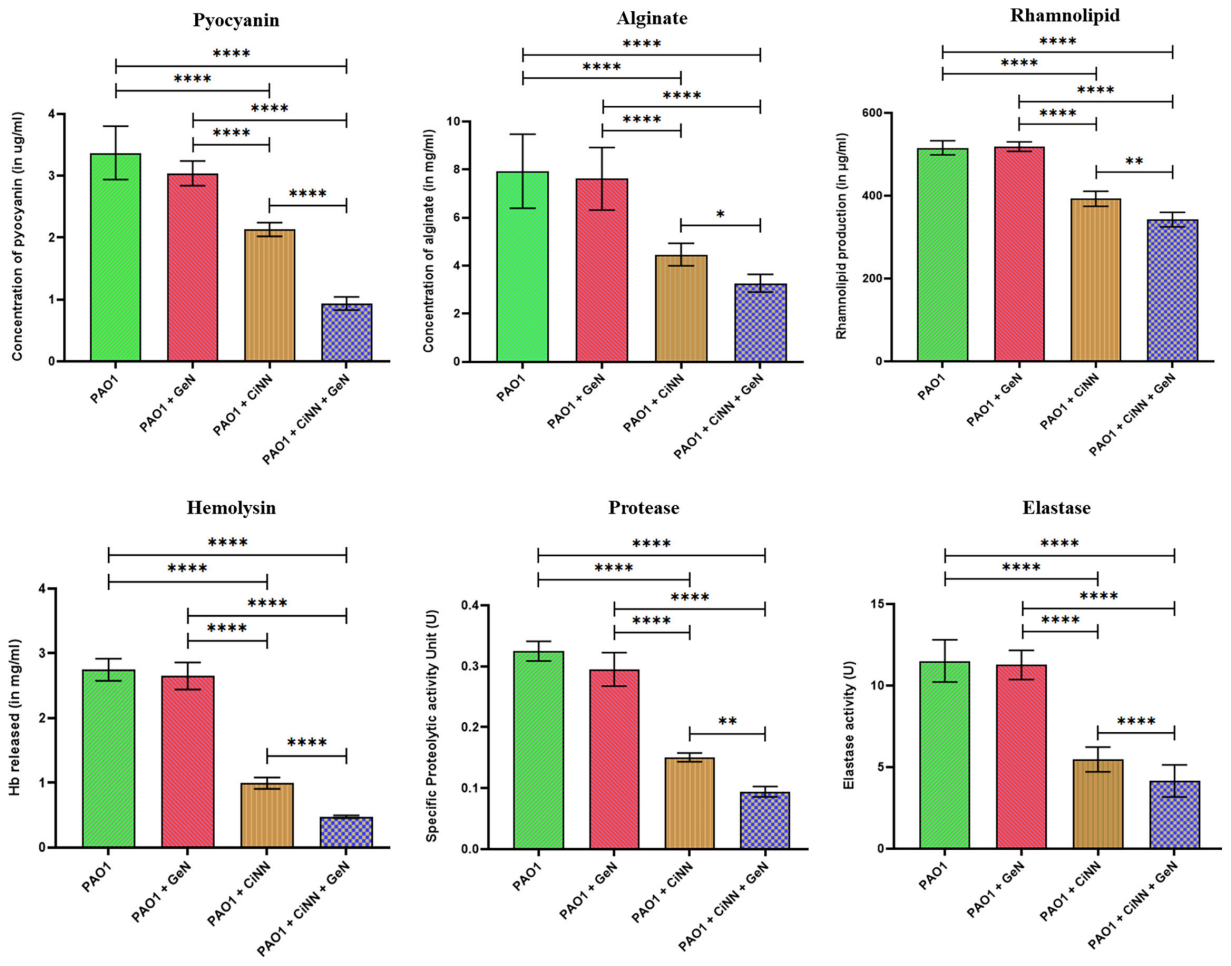
Anti-virulence effects of sub-MICs of CiNN and GeN against *P. aeruginosa* PAO1, alone and in combination. Various QS-regulated virulence factors including, pyocyanin, alginate, rhamnolipid, hemolysin, protease, and elastase production were assayed in the presence and absence of the drugs. (*p ≤ 0.05, **p ≤ 0.01, ****p ≤ 0.0001).

The virulence of *P. aeruginosa* is driven by the production of diverse QS-regulated virulence determinants ranging from cell-associated host colonization factors to destructive extracellular enzymes, toxins, and toxic secondary metabolites. Standard assays were performed to examine the levels of various QS-regulated virulence factors of *P. aeruginosa*. Production of all the test virulence factors (alginate, rhamnolipids, pyocyanin, and hemolysin) and enzyme activities (elastase, protease) were significantly impeded in the presence of CiNN alone, while combinational treatment with GeN exerted a greater inhibitory effect (**Figure 6**). CiNN alone reduced the production of pyocyanin, alginate, rhamnolipids, and hemolysin by 1.58- (2.13 μg/ml), 1.78- (4.46 mg/ml), 1.31- (392.54 μg/ml), and 2.76-folds (0.997 mg/ml Hb released), while in combination with GeN, the virulence factors were further diminished by 3.61- (0.935 μg/ml), 2.43- (3.27 mg/ml), 1.51- (342.3 μg/ml), and 5.76-folds (0.477 mg/ml Hb released), respectively as compared to the untreated control. Elastase and protease enzyme activities were also suppressed with CiNN alone (by 2.10- and 2.16-folds) and the drug combination (2.77- and 3.44-folds), respectively. In line with our previous findings, GeN alone did not inhibit the production of any pseudomonal virulence factor. Altogether, our results suggest that GeN boosts the QQ potential of CiNN, thereby suppressing the production of virulence factors in *P. aeruginosa*.

### 3.8 CiNN Abrogates Biofilm Formation in *P. aeruginosa* by Inhibiting EPS Production

**Figure 7:**
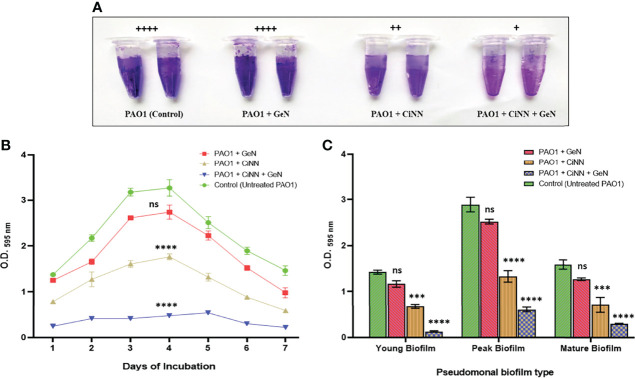
Examining the anti-fouling effect of CiNN and GeN, alone and in combination against *P. aeruginosa* PAO1. **(A)** Qualitative biofilm formation assay in microcentrifuge tubes. Adherence scores have been mentioned above in each experimental group. (+): weak adherence, (++): moderate adherence, (+++): strong adherence, and (++++): very strong adherence. **(B)** Biofilm inhibition pattern of PAO1 in the presence and absence of CiNN and GeN obtained from quantitative CV assay. **(C)** Comparative analysis of eradication ability of CiNN and GeN, alone and in combination against preformed biofilms of PAO1 of different ages. (ns, not significant, ***p ≤ 0.001, ****p ≤ 0.0001).

**Figure 8:**
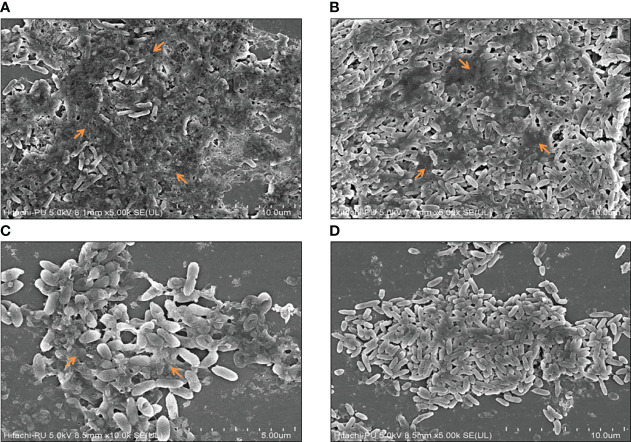
Microscopic visualization of peak day pseudomonal biofilms in the presence and absence of the test drugs using FESEM. **(A)** Untreated control, **(B)** GeN-treated culture, **(C)** CiNN-treated culture, and **(D)** CiNN-GeN-treated culture. The arrows indicate EPS production within the PAO1 biofilms.

Biofilm formation was assessed in the presence and absence of sub-MICs of the drugs by qualitative and quantitative assays coupled with microscopic examination. Preliminary experiments revealed the anti-biofilm prospects of CiNN alone and in combination with GeN (**Figure 7A**). Untreated PAO1 and GeN alone demonstrated strong biofilm-forming abilities (denoted by ++++) characterized by the intense color of CV, while CiNN resulted in the formation of a moderate biofilm (++). Interestingly, combinational treatment with CiNN and GeN resulted in the development of a weaker biofilm (+) compared to the untreated control. This provided a strong basis to explore the anti-fouling prospects of the drug combination. Further, the biofilm-forming ability of PAO1 was quantitated over seven days in the presence and absence of sub-MICs of the drug. *P. aeruginosa* PAO1 demonstrated strong biofilm-forming abilities that gradually increased with incubation time, attaining a peak on the 4^th^ day followed by a decline phase which was indicative of biofilm maturation and dispersion (**Figure 7B**). Biofilm formation in PAO1 showed a notable inhibition in the presence of CiNN alone (~ 46% on peak day) as compared to the untreated control, while it remained unaffected with GeN alone. However, biofilm production was nearly abrogated (~ 85.2%) upon exposure with CiNN and GeN in combination on the peak day, strongly pointing toward the fact that GeN could enhance the anti-fouling properties of CiNN when used in combination. Further, the effect was scrutinized over preformed pseudomonal biofilms of different ages (young, peak, and mature biofilms). Interestingly, the CiNN-GeN combination eradicated preformed PAO1 biofilms of all test groups (young: 90.54%, peak: 78.8%, and mature: 81.2%) with a significantly higher potency than CiNN alone (young: 52.4%, peak: 53.9%, and mature: 55.1%), as compared to the untreated control that displayed strong biofilm formation abilities (**Figure 7C**). Establishing a correlation with our previous findings, GeN alone was ineffective in eradicating the preformed biofilms of PAO1. Hence, it was concluded that the drug combination was capable of inhibiting biofilm formation and eradicating preformed biofilms of *P. aeruginosa* PAO1 *in vitro*. To confirm these experimental findings, PAO1 biofilms grown in the presence and absence of the test agents (alone and in combination) on glass coverslips were visualized using FESEM. Untreated PAO1 showed extensive biofilm formation with bacterial cells embedded in a dense layer of EPS (**Figure 8A**), similar to that of cells treated with sub-MIC of GeN alone (**Figure 8B**). Interestingly, treatment with CiNN did not eliminate bacterial growth but induced a change in the biofilm architecture by inhibiting EPS production (**Figure 8C**). Cells cultured in the presence of the CiNN-GeN combination showed significant inhibition of biofilm and displayed features with near negligible EPS production (**Figure 8D**). Taken together, our findings suggest that CiNN inhibits biofilm formation in *P. aeruginosa* PAO1 by abrogating EPS production, which in turn gets enhanced when supplemented in combination with GeN.

### 3.9 GeN Enhances the Protective Effect of CiNN and Increases the Survival Rate of *C. elegans* Following PAO1 Challenge

**Figure 9:**
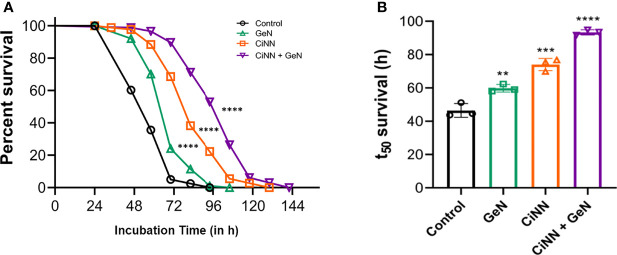
Anti-virulence effect of CiNN and GeN against PAO1 infection in *C. elegans*. **(A)** Percent survival of PAO1-infected *C. elegans* from slow-killing assays in the presence and absence of CiNN and GeN at sub-MICs, alone and in combination. **(B)** Comparative analysis of worm lifespan in terms of t_50_ survival values following drug treatment (**p ≤ 0.01, ***p ≤ 0.001, ****p ≤ 0.0001).

To validate the anti-virulent potential of CiNN, slow-killing assays were performed on *C. elegans* with *P. aeruginosa* PAO1 on NGM plates supplemented with the drugs at sub-MICs, individually and in combination. *C. elegans* infected with PAO1 (control) exhibited nearly 100% mortality at 84 h. The percentage survival of PAO1-infected *C. elegans* at 48, 60, and 72 h was found to be 59%, 36%, and 8%, respectively (**Figure 9A**). Interestingly, CiNN supplementation increased the worm survival significantly by 98%, 68%, and 23% at 48, 72, and 96 h, respectively (**Figure 9A**). While GeN alone did not rescue worms from PAO1 infection, the mortality rate was similar to that of untreated PAO1. In addition, treatment with the GeN-CiNN combination demonstrated increased survival rates of *C. elegans*. The percentage survival following combinational drug treatment was found to be 99%, 91%, and 52% at 48, 72, and 96 h, respectively (**Figure 9A**). Also, the time course at which only 50% of the worms survived (t_50_) was calculated using the survival curves. The t_50_ value was found to be 44.5 h with PAO1 (control) which increased drastically following drug treatment (**Figure 9B**). GeN alone was able to improve the t_50_ value to 59 h, but at a lower level of significance (p ≤ 0.01). Interestingly, the lifespan of *C. elegans* was significantly enhanced in the presence of CiNN alone (t_50_ = 75.5 h; p ≤ 0.001), while treatment with the drug combination improved worm survival and increased t_50_ value by more than two folds (t_50_ = 93.5 h; p ≤ 0.0001) (**Figure 9B**). Hence, the results indicated the protective nature of the CiNN-GeN combination because of its ability to rescue *C. elegans* from *P. aeruginosa* infection.

## 4 Discussion

The pathogenicity of *P. aeruginosa* primarily depends on the production of diverse virulence factors, including destructive enzymes, secondary metabolites, toxins, siderophores, and resilient biofilms that are stringently regulated by its intricate QS mechanisms (Moradali et al., 2017). The last decade has seen an unprecedented increase in AMR globally, underscoring the need for developing alternative intervention strategies to existing antimicrobial regimens (Khullar et al., 2022). In this direction, targeting the bacterial QS systems to attenuate pathogen virulence has gained momentum amongst scientists, paving the way for innovating novel anti-virulence therapies (Chadha et al., 2021a). Natural phytoproducts like EOs and their bioactive constituents have exhibited promising anti-QS activities, thereby attenuating the virulence of *P. aeruginosa* (Chadha et al., 2021a). Niu et al. pioneered studies on CiNN and demonstrated its ability to interfere with QS in *Vibrio harveyi* by modulating the transcription of QS genes (Niu et al., 2006). Also, reports briefly highlighting the QQ potential of cinnamon oil and it’s principal bioactive, cinnamaldehyde, against *P. aeruginosa* have surfaced recently (Ahmed et al., 2019; Topa et al., 2020). However, these findings need further validation to elucidate the precise mechanism behind the anti-QS activity of CiNN and its anti-virulent potential *in vivo*. Hence, the present study focused on evaluating the QQ activity and anti-virulent potential of CiNN alone and in combination with GeN against *P. aeruginosa* at sub-lethal concentrations.

**Figure 10:**
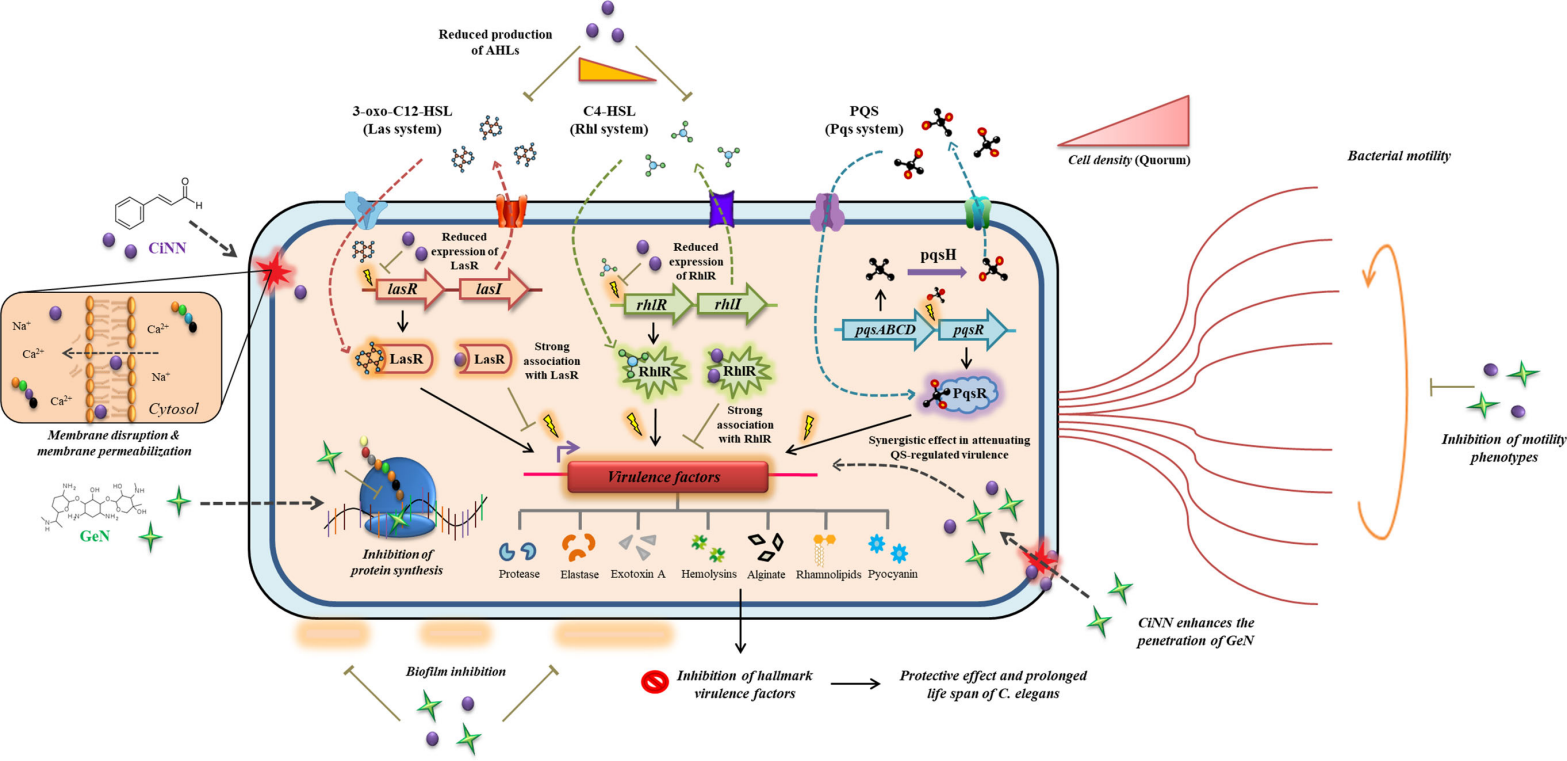
Mechanistic model depicting the possibilities behind the synergistic interaction of CiNN-GeN and its outcomes in attenuating the QS circuits and QS-regulated virulence in *P. aeruginosa*. The distinct mode of action exhibited by CiNN and GeN have been illustrated alongside their combination activity.

We initiated our studies by determining the MICs of CiNN and GeN, followed by assessing the impact of sub-MICs on the growth profile of *P. aeruginosa* PAO1. Previous reports have indicated that sub-MICs of cinnamon oil and *C. tamala* oil alter the growth kinetics of *P. aeruginosa* in a dose-dependent manner (Leoni et al., 2015; Farisa Banu et al., 2017). Our findings were consistent with the previous reports indicating the antimicrobial activity of CiNN and GeN at higher concentrations and anti-QS activity of CiNN at lower concentrations. Subsequently, we determined the type of association shown between CiNN and GeN against PAO1. Based on the results from chequerboard assays, the combined use of CiNN and GeN revealed a synergistic activity, which was further substantiated by time-kill kinetics assays. Recent studies have also confirmed the synergistic interaction of CiNN in combination with colistin (Topa et al., 2020) and ciprofloxacin (Kart et al., 2021). The synergistic interaction between the drugs may be attributed to their distinct modes of action, with CiNN causing membrane disruption along with oxidative damage (Doyle and Stephens, 2019) and GeN inhibiting bacterial protein synthesis (He et al., 2018). This makes the bacterial cell more susceptible since the drugs can act in a concerted manner at two distinct cellular fronts. This exemplary ‘multi-target synergy approach’ has recently drawn attention for its possible role in attenuating bacterial virulence to treat *P. aeruginosa* infections in cystic fibrosis patients (Shaw and Wuest, 2020). Secondly, increased membrane permeabilization by CiNN (Topa et al., 2018) may facilitate easy penetration of GeN into the bacterial cell, thereby compromising the translation machinery associated with the production of different virulence factors. These possible interactions may also be supplemented by the QQ potential of CiNN due to structural similarities with the AHL molecules, 3-oxo-C12-HSL and C4-HSL, thereby attenuating QS circuits, abrogating biofilm formation and ultimately altering bacterial virulence and fitness. Taken together, these speculations can provide the basis for the synergistic interaction between CiNN and GeN. Based on these possibilities; we also propose a model for this drug interaction illustrating the final outcomes of CiNN-GeN treatment on the virulence of *P. aeruginosa* (**Figure 10**).

The virulence arsenal of *P. aeruginosa* is expressed following the induction of QS circuits, Las, Rhl, and Pqs in a hierarchical manner. The Las system regulates the expression of genes related to hemolysins, proteases, elastases, and pseudomonal biofilm production (Chadha et al., 2021a), while the Rhl pathway drives the expression of virulence genes associated with bacterial motility and production of pyocyanin, hydrogen cyanide, siderophores, elastases, and alkaline protease (Papenfort and Bassler, 2016). On the other hand, the Pqs system regulates the genes involved in biofilm formation, promoting swimming motility and production of pyocyanin, proteases, elastases, rhamnolipids, and siderophores (García-Reyes et al., 2020). Thus, QS becomes a quintessential target for attenuating pseudomonal virulence since it would directly suppress the virulence factors without posing much selection pressure. Hence, we probed the anti-QS prospects of this novel phytochemical-antibiotic combination using the *A. tumefaciens* NTL4 biosensor strain. This strain has been genetically engineered to mediate the AHL-induced expression of β-galactosidase (*lacZ*), resulting in the enzymatic hydrolysis of X-gal (5-Bromo-4-chloro-3-indolyl-β-D-galactopyranoside) and the development of blue coloration (Bose et al., 2020). Agents with anti-QS activity prevent the activation of *lacZ*, eventually yielding bacterial growth with white coloration. Notably, CiNN displayed efficient QQ activity, which got augmented with GeN supplementation, as evident from an increase in the diameter of the colorless turbid zone around wells. Preceding studies have also established that although GeN does not possess anti-QS properties, still, it can complement the QQ potential of vitexin, inhibiting the production of virulence factors and biofilm formation in *P. aeruginosa* (Das et al., 2016).

Intrigued by this finding, we further assayed the level of AHL production in PAO1 following CiNN and GeN exposure at sub-MICs. The agar overlay method has been widely used and considered reliable for the qualitative detection of AHLs in the form of colored spots (Gui et al., 2018). Reduction in the intensity of blue spots of AHLs on TLC plates provided us the first clue suggesting that CiNN alone and in combination with GeN reduces 3-oxo-C12-HSL and C4-HSL production. To extend this finding, we quantified the level of AHL production in drug-treated and untreated supernatants of PAO1 in terms of β-galactosidase activity (in MU) resulting from AHL-mediated induction of *lacZ* on pZLR4 in *A. tumefaciens* NTL4. Since AHL molecules directly drive the expression of β-galactosidase, the enzyme activity shows a linear correlation with the amount of AHL produced. Results of the β-galactosidase assay were consistent with the findings of the agar overlay experiment, indicating a significant reduction in β-galactosidase activity following combinational treatment. This confirmed our speculation revealing that GeN elevates the anti-QS potential of CiNN by inhibiting the synthesis of AHLs in *P. aeruginosa* PAO1. Motivated by this, qRT-PCR was performed to analyze the transcript levels of various QS and virulence genes under test and control conditions. Our results were in accordance with erstwhile reports (Ahmed et al., 2019), illustrating a significant downregulation in the expression levels of *las* and *rhl* QS genes and virulence genes involved in rhamnolipid (*rhlAB*), alkaline protease (*aprA*), exotoxin A (*toxA*), and hemolysin (*plcH*) production following treatment with CiNN alone and in combination with GeN. The latter enhanced the repression exerted by CiNN over the expression of QS genes, which was subsequently validated by *in silico* analysis and estimating the production of downstream virulence factors in drug-treated PAO1 cultures.

Computational analysis provides an excellent tool to simulate the ligand-receptor interactions at the atomic level (Kumar et al., 2021). Hence, *in silico* analysis was carried out to gain better insights into the possible associations between CiNN and the QS receptors of *P. aeruginosa*. Considering the relatively small size of CiNN, it can easily gain access to the active site of the QS receptors. Molecular docking also supported this hypothesis and CiNN was predicted to strongly associate with the active sites of the QS receptors. The binding energies revealed that CiNN exhibits the highest affinity towards LasR (-12.0 kcal/mol), followed by PqsR (-8.2 kcal/mol) and RhlR (-7.6 kcal/mol). This data could be easily correlated with the results from qRT-PCR analysis, which indicated the highest fold reduction in the transcript levels of LasR following CiNN treatment. Moreover, the findings could be related to the molecular structure of CiNN, which resembles that of 3-oxo-C12-HSL and C4-HSL, bearing a homocyclic ring structure and AHL-like short side chain with different levels of oxygenation (Chadha et al., 2021a). This structural similarity can be accounted for the strong affinity predicted between CiNN and the QS receptors, imparting stability to the ligand-protein complex as a consequence of which CiNN may have silenced QS circuits in *P. aeruginosa*. Nevertheless, this warrants further validation using high-throughput techniques assisted with molecular simulation studies.

We further examined the anti-motility effect of the drug combination against *P. aeruginosa.* Apart from its role in promoting surface colonization and biofilm formation, motility enables bacteria to resist flow, host defenses, and also compete with other pathogenic varieties in polymicrobial niches by disseminating itself in the host tissues (Chadha et al., 2021b). The motility phenotypes of *P. aeruginosa* are mediated by bacterial flagella and type IV pilus. Studies have implicated the role of type IV pilus in regulating the swarming and twitching motility of this pathogen (Daniels et al., 2004; Kazmierczak et al., 2015). However, QS has been shown to modulate both swimming and swarming motilities of *P. aeruginosa* (Daniels et al., 2004; Williams and Cámara, 2009). On the contrary, reports have suggested that twitching is not regulated by QS (Beatson et al., 2002). Consistent with the previous reports on cinnamon oil (Leoni et al., 2015) and CiNN (Topa et al., 2018), all the three motility phenotypes were significantly inhibited by CiNN alone and in combination with GeN. The inhibition of swimming and swarming motility in *P. aeruginosa* can be attributed to the anti-QS properties of CiNN, which further got enhanced in the presence of GeN. Unlike the other two motilities, twitching motility is mediated by successive extension and retraction of type IV pili (Mattick, 2002). Since CiNN inhibited swarming and twitching motilities, both of which are modulated by type IV pili, it may be inferred that CiNN interferes with the functions of the type IV pilus. Moreover, a recent report highlighted that *P. aeruginosa* directs twitching by sensing mechanical input generated by the type IV pili (Kühn et al., 2021). Hence, it may be speculated that CiNN abrogates the mechanotactic functions of type IV pilus, thereby inhibiting twitching motility in *P. aeruginosa*. This firmly substantiated our earlier findings and provided fertile grounds to assess the impact of this combination on the production of key pseudomonal virulence factors. In accordance with our initial results, the hallmark virulence factors, including alginate, rhamnolipids, pyocyanin, hemolysins, elastases, and proteases were found to be significantly inhibited in the presence of CiNN as compared to the untreated control, while supplementing GeN enhanced the anti-virulence potential of CiNN. This inhibitory effect can be attributed to the enhanced QQ activity of the CiNN-GeN combination resulting from the downregulation of QS genes and subsequent reduction in AHL production in *P. aeruginosa* PAO1.

Biofilm formation in *P. aeruginosa* has been regarded as a crucial virulence hallmark that imparts resistance against phagocytosis, oxidative stress, nutrient depletion, and penetration of antimicrobial drugs (Moradali et al., 2017). All the three QS circuits play an important role in transitioning between the different stages of biofilm formation and hence provide an opportunity to repurpose QQ drugs as potent anti-biofilm agents. The extracellular polymeric substance (EPS) of pseudomonal biofilms constitutes glycoprotein, glycolipids (rhamnolipids), polysaccharides (alginate, Pel, and Psl), and extracellular DNA. Since the production of alginate and rhamnolipids was found to be significantly reduced in the presence of drug combinations, we speculated that this might directly modulate biofilm formation in PAO1. Our results demonstrated the anti-fouling prospects of CiNN in combination with GeN by manipulating biofilm architecture. The FESEM micrographs provided conclusive evidence that CiNN treatment abrogates EPS production, resulting in delicate biofilms with reduced adherence. A notable reduction in alginate and rhamnolipid production coincided with inhibition of EPS production in drug-treated cultures. The presence of bacteria in the exopolysaccharide matrix has been associated with the transition from reversible to irreversible attachment during biofilm development (Moradali et al., 2017). This drug combination also eradicated young, mature, and old preformed pseudomonal biofilms. The existing literature substantiates our findings on inhibition and eradication of biofilms by CiNN, which has been previously implicated with a reduction in the intracellular levels of c-di-GMP, a signaling molecule associated with biofilm formation (Topa et al., 2018). Hence, the CiNN-GeN combination harbors profuse QQ activity and also demonstrates potent anti-fouling properties against *P. aeruginosa*.

To validate the anti-virulence properties of this drug combination, a *C. elegans* infection model that has been widely used to examine the anti-infective properties of compounds against *P. aeruginosa* was employed (Bajire et al., 2021). In slow-killing assays, PAO1 resulted in 100% mortality at 84 h. In comparison, the drug combination protected *C. elegans* from PAO1 infection and enhanced survival rates by 52% at 96 h. *P. aeruginosa*-mediated mortality in worms has been associated with cyanide toxicity caused by hydrogen cyanide (Tan et al., 1999), the product of *hcn* operon that is stringently regulated by the LasR and RhlR QS regulators (Pessi and Haas, 2000). Previous studies have implicated the role of QQ phytochemicals, including 6-methylcoumarin (Bajire et al., 2021), curcumin (Rudrappa and Bais, 2008), and cinnamic acid (Rajkumari et al., 2018), in downregulating the QS-associated virulence (enzymes and toxic metabolites) and protecting *C. elegans* from PAO1 infection, thereby enhancing worm survival. The protective role of CiNN in combination with GeN can be strongly associated with its enhanced anti-QS, anti-virulent, and anti-biofilm properties. CiNN individually also displayed similar effects but at a relatively lower magnitude. Moreover, the drug combination remarkably increased the lifespan of *C. elegans* which was evident by a nearly two-fold increase in the t_50_ value (93.5 h), as compared to the untreated control (44.5 h). Hence, our findings reinforce the application of CiNN as a potent anti-QS phytochemical to combat pseudomonal infections. Nevertheless, combinational therapy with antibiotics can certainly improve the therapeutic outcome and the quality of patient life, thereby reducing the risk of developing AMR. In this direction, further investigations are warranted to precisely delineate the microbial pathways or systems for absolute inhibition of virulence mechanisms in other animal models to ensure complete protection from pseudomonal infections.

## 5 Conclusion

Based on the experimental findings from our study, we provide the first insights into the molecular mechanism behind the anti-QS activity of CiNN. We conclude that sub-MIC of CiNN effectively silences the QS circuits in *P. aeruginosa* by downregulating QS genes and abrogating the biosynthesis of AHL molecules. The QQ potential of CiNN was attributed to its ability to strongly interact with the QS receptors of *P. aeruginosa*. This, in turn, attenuates the QS-associated virulence factors and biofilm formation, rendering the pathogen avirulent. Moreover, supplementing GeN enhances the QQ activity and augments the anti-virulent potential of CiNN. This novel combination also improves the ability of *C. elegans* to survive pseudomonal infection. Hence, CiNN proves to be a potent anti-virulent phytochemical that can be valuable for developing novel intervention strategies against *P. aeruginosa* in combination with antibiotics. However, detailed investigations are further warranted to evaluate its therapeutic efficacy using *in vivo* models to exploit its anti-virulent potential in clinical practices in the future.

## Data Availability Statement

The original contributions presented in the study are included in the article/**Supplementary Material**. Further inquiries can be directed to the corresponding author.

## Author Contributions

JC: experimental design and initiation, methodology, data analysis, execution (*in vitro* and *in silico*), and manuscript writing. R: performed slow killing assays with *C. elegans* and data analysis. JS: supervision of *C. elegans*-related experiments, data analysis, data curation, and manuscript editing. SC: motivation, critical analysis, and supervision involved in the study design and execution. KH: idea conceptualization, supervision of the study, continuous motivation, data analysis and curation, critical analysis, and editing of the manuscript. All authors read and approved the final version of this manuscript.

## Conflict of Interest

The authors declare that the research was conducted in the absence of any commercial or financial relationships that could be construed as a potential conflict of interest.

## Publisher’s Note

All claims expressed in this article are solely those of the authors and do not necessarily represent those of their affiliated organizations, or those of the publisher, the editors and the reviewers. Any product that may be evaluated in this article, or claim that may be made by its manufacturer, is not guaranteed or endorsed by the publisher.

## Supplementary Material

Supplementary Figure 1 Resazurin dye reduction assay for determining the MICs of CiNN and GeN against *P. aeruginosa* PAO1.

Supplementary Figure 2 Molecular docking analysis predicting the probable interactions between CiNN and various QS receptors of *P. aeruginosa* using AutoDock Vina (Version 1.2.0).

Supplementary Figure 3 Molecular docking analysis predicting the probable interactions between Furanone C 30 (control) and various QS receptors of *P. aeruginosa* using AutoDock Vina (Version 1.2.0).

## Abbreviations

3-oxo-C12-HSL, N-3-oxo-dodecanoyl-L-Homoserine lactone; AHL, acyl-homoserine lactone; AMR, antimicrobial resistance; C4-HSL, N-butanoyl-L-homoserine lactone; CiNN, cinnamaldehyde; EO, essential oil; FIC, Fractional inhibitory concentration; FICI, FIC index; GeN, gentamicin; MeOH, methanol; MIC, minimum inhibitory concentration; PBS, phosphate buffer saline; QQ, quorum quenching; QS, quorum sensing; X-gal, 5-Bromo-4-chloro-3-indolyl-β-D-galactopyranoside.
