# Supplementary figures and images for: Gentamicin Augments the Quorum Quenching Potential of Cinnamaldehyde In Vitro and Protects Caenorhabditis elegans From Pseudomonas aeruginosa Infection
Source: Front Cell Infect Microbiol. 2022 Jun 15;12:899566. doi: 10.3389/fcimb.2022.899566 (PMC9240785; doi:10.3389/fcimb.2022.899566)

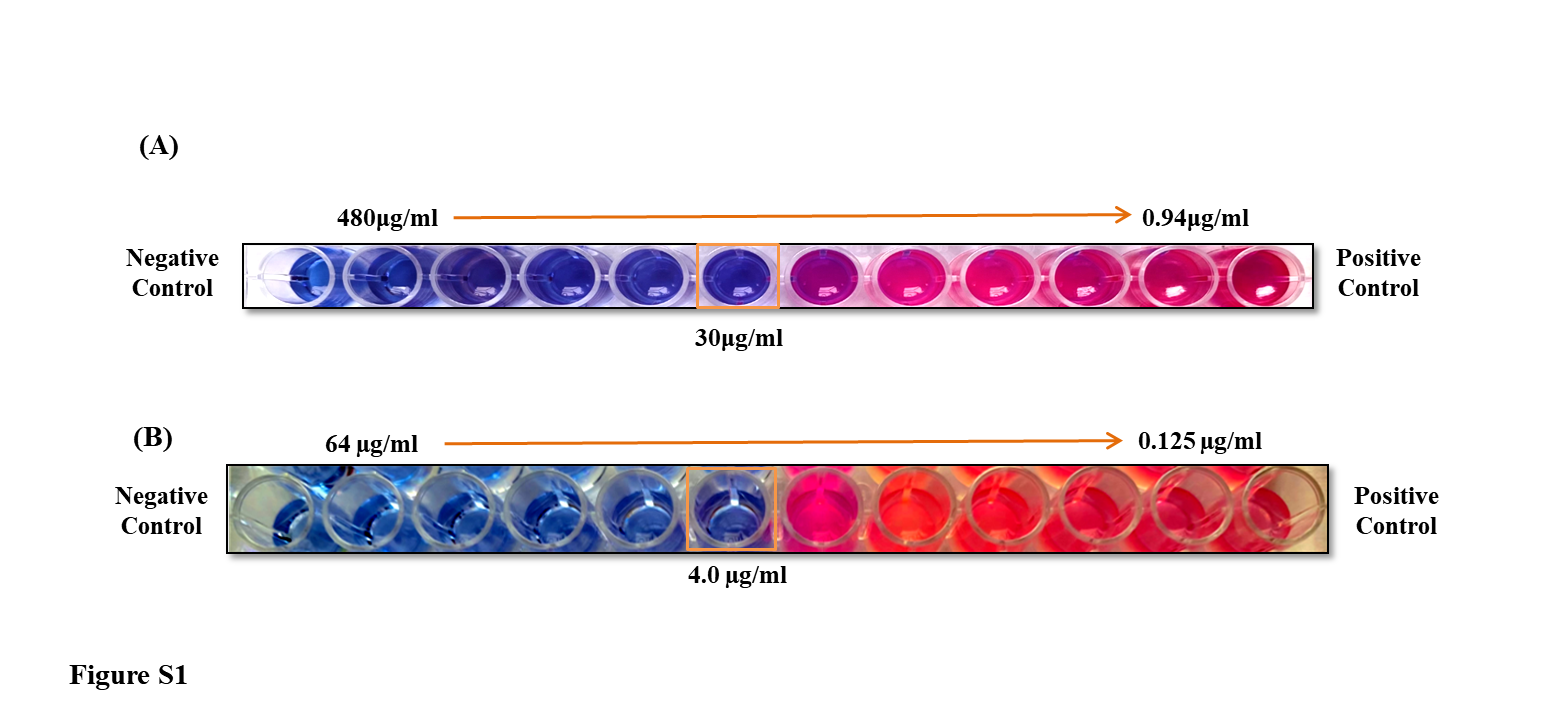

Supplement: Supplementary Figure 1 — Resazurin dye reduction assay for determining the MICs of CiNN and GeN against P. aeruginosa PAO1. [file Image_1.tif]

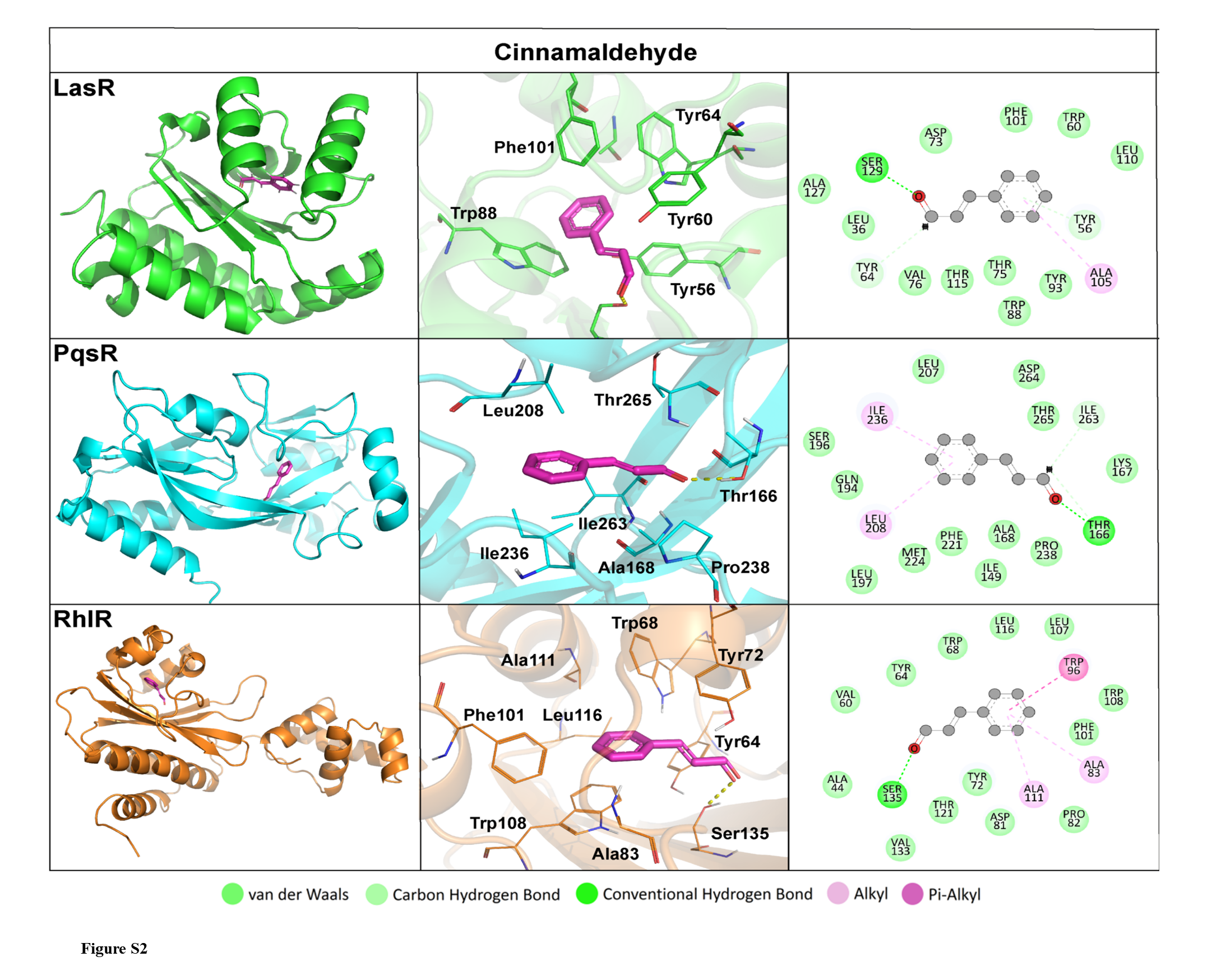

Supplement: Supplementary Figure 2 — Molecular docking analysis predicting the probable interactions between CiNN and various QS receptors of P. aeruginosa using AutoDock Vina (Version 1.2.0). [file Image_2.tif]

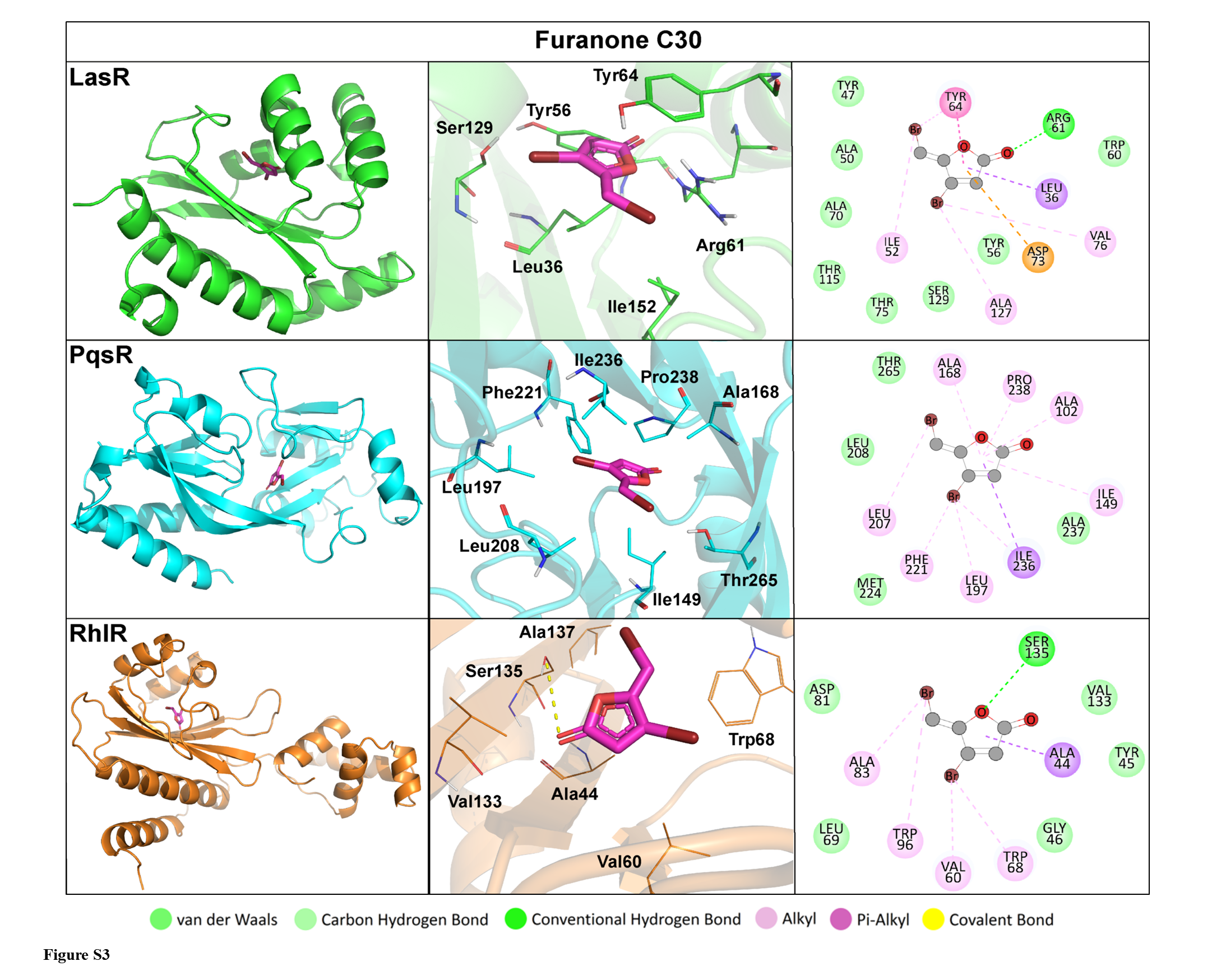

Supplement: Supplementary Figure 3 — Molecular docking analysis predicting the probable interactions between Furanone C 30 (control) and various QS receptors of P. aeruginosa using AutoDock Vina (Version 1.2.0). [file Image_3.tif]
