# Supplementary material for: Gentamicin Augments the Quorum Quenching Potential of Cinnamaldehyde In Vitro and Protects Caenorhabditis elegans From Pseudomonas aeruginosa Infection
Source: Front Cell Infect Microbiol. 2022 Jun 15;12:899566. doi: 10.3389/fcimb.2022.899566 (PMC9240785; doi:10.3389/fcimb.2022.899566)
Supplement: Supplementary file 4 [file Table_1.doc]

| **Target Gene** | **Primer sequence (5´-3´)** | **Amplicon size (in bp)** |
| --- | --- | --- |
| ***lasI*** | *lasI*-Fwd: CGTGCTCAAGTGTTCAAGG  *lasI*-Rev: TACAGTCGAAAAGCCCAG | 295 |
| ***lasR*** | *lasR*-Fwd: AAGTGGAAAATTGGAGTGGAG  *lasR*-Rev: GTAGTTGCCGACGACGATGAAG | 130 |
| ***rhlI*** | *rhlI*-Fwd: TTCATCCTCCTTTAGTCTTCCC  *rhlI*-Rev: TTCCAGCGATTCAGAGAGC | 155 |
| ***rhlR*** | *rhlR*-Fwd: TGCATTTTATCGATCAGGGC  *rhlR*-Rev: CACTTCCTTTTCCAGGACG | 133 |
| ***rhlAB*** | rhlAB-Fwd: TCATGGAATTGTCACAACCGC  rhlAB-Rev: ATACGGCAAAATCATGGCAAC | 151 |
| ***toxA*** | toxA-Fwd: GGAGCGCAACTATCCCACT  toxA-Rev: TGGTAGCCGACGAACACATA | 150 |
| ***plcH*** | plcH-Fwd: GAAGCCATGGGCTACTTCAA  plcH-Rev: AGAGTGACGAGGAGCGGTAG | 307 |

**Table S1. Details of primers used for quantifying the relative gene expression of QS and virulence genes of *P. aeruginosa* PAO1.**

| **QS Receptors** | **Ligands** | **Binding Energy (kcal/mol)** | **Interacting amino acid residues** |
| --- | --- | --- | --- |
| **LasR** | **3-oxo-C12-HSL** | -13.4 | Leu36, Try56, Trp60, Try64, Asp73, Val76, Cys79, Try93, Phe101, Ala105, Ser129 |
|  | **CiNN** | -12.0 | **Leu36**, **Tyr56**, **Trp60**, **Tyr64**, **Asp73**, Thr75, **Val76**, Trp88, **Tyr93**, **Phe101**, **Ala105**, Leu110, **Thr115**, **Ala127**, **Ser129** |
|  | **Furanone C-30** | -8.9 | Leu36, Tyr47, Ala50, Ile52, Tyr56, Trp60, Arg61, Tyr64, Ala70, Asp73, Thr75, Val76, Thr115, Ala127, Ser129 |
| **RhlR** | **C4-HSL** | -6.4 | Ser24, Val27, Try45, His61, Leu166, Thr167, Leu169, Glu170 |
|  | **CiNN** | -7.6 | **Ala44**, **Val60**, Tyr64, **Trp68**, Tyr72, Asp81, Pro82, **Ala83**, **Trp96**, Phe101, Leu107, Trp108, Ala111, Leu116, Thr121, **Val133**, **Ser135** |
|  | **Furanone C-30** | -6.43 | Ala44, Tyr45, Gly46, Val60, Trp68, Leu69, Asp81, Ala83, Trp96, Val133, Ser135 |
| **PqsR** | **PQS** | -9.1 | Pro129, Ala130, Ile149, Ala168, Leu197, Leu207, Leu208, Phe221, Ile236, Ala237, Pro238, Thr265 |
|  | **CiNN** | -8.2 | Gln104, **Ile149**, Thr166, Lys167, **Ala168**, Ser196, **Leu197**, **Leu207**, **Leu208**, **Phe221**, **Met224**, **Ile236**, Pro238, Ile263, Asp264, **Thr265** |
|  | **Furanone C-30** | -7.8 | Ala102, Ile149, Ala168, Leu197, Leu207, Leu208, Phe221, Met224, Ile236, Ala237, Pro238, Thr265 |

**Table S2.** Predicted interactions of natural ligands, CiNN, and furanone C-30 with LasR, RhlR, and PqsR QS receptors in terms of binding energy. Boldface amino acid residues of CiNN were found to have overlapping interactions with that of either the natural ligand of each QS receptor or furanone C-30.
